# Species-Specific Quality Control, Assembly and Contamination Detection in Microbial Isolate Sequences with AQUAMIS

**DOI:** 10.3390/genes12050644

**Published:** 2021-04-26

**Authors:** Carlus Deneke, Holger Brendebach, Laura Uelze, Maria Borowiak, Burkhard Malorny, Simon H. Tausch

**Affiliations:** Department Biological Safety, German Federal Institute for Risk Assessment, 10589 Berlin, Germany; Carlus.Deneke@bfr.bund.de (C.D.); holger.brendebach@bfr.bund.de (H.B.); laura.uelze@bfr.bund.de (L.U.); maria.borowiak@bfr.bund.de (M.B.); Burkhard.Malorny@bfr.bund.de (B.M.)

**Keywords:** whole genome sequencing, next generation sequencing, quality control, isolate sequencing, pipeline, assembly, contamination, reproducibility, interoperability

## Abstract

Sequencing of whole microbial genomes has become a standard procedure for cluster detection, source tracking, outbreak investigation and surveillance of many microorganisms. An increasing number of laboratories are currently in a transition phase from classical methods towards next generation sequencing, generating unprecedented amounts of data. Since the precision of downstream analyses depends significantly on the quality of raw data generated on the sequencing instrument, a comprehensive, meaningful primary quality control is indispensable. Here, we present AQUAMIS, a Snakemake workflow for an extensive quality control and assembly of raw Illumina sequencing data, allowing laboratories to automatize the initial analysis of their microbial whole-genome sequencing data. AQUAMIS performs all steps of primary sequence analysis, consisting of read trimming, read quality control (QC), taxonomic classification, de-novo assembly, reference identification, assembly QC and contamination detection, both on the read and assembly level. The results are visualized in an interactive HTML report including species-specific QC thresholds, allowing non-bioinformaticians to assess the quality of sequencing experiments at a glance. All results are also available as a standard-compliant JSON file, facilitating easy downstream analyses and data exchange. We have applied AQUAMIS to analyze ~13,000 microbial isolates as well as ~1000 in-silico contaminated datasets, proving the workflow’s ability to perform in high throughput routine sequencing environments and reliably predict contaminations. We found that intergenus and intragenus contaminations can be detected most accurately using a combination of different QC metrics available within AQUAMIS.

## 1. Introduction

Whole-genome sequencing (WGS) has become the new gold standard for the analysis of bacterial isolates. The high-resolution method WGS allows the investigation and comparison of microbial genome sequences on a nucleotide level. Importantly, WGS methods facilitate the detection of any gene of interest, genomic differences at single base pair level and phylogenetic analyses [1]. The broad applicability, combined with the high resolution power, have led to the implementation of WGS in many different research fields, including clinical and public health settings [2]. In recent years, the steady drop in sequencing costs has enabled more and more laboratories to acquire sequencing devices for routine sequencing. However, a successful implementation of WGS technology requires not only proper handling of materials, instruments and protocols, but also the processing of the resulting sequence data. As WGS generates unprecedented amounts of data, many laboratories find themselves challenged by the need to store, process and analyze these data. Crucially, a stringent quality control and an optimal pre-processing of the raw sequencing data are required to ensure the integrity of any downstream analysis [3]. In the best case, a sophisticated IT infrastructure and staff with in-depth bioinformatic and IT expertise are available for these tasks. However, many institutions lack these resources and as a result, the integrity of the data may be compromised, sequencing data may not be analyzed correctly or there may be a backlog of analyses and substantial delays in receiving results. It is therefore extremely important to provide non-bioinformaticians with robust and easy-to-use tools, which allow them to perform primary analyses self-sufficiently and to access and interpret results independently [4].

A robust quality control is especially important, when laboratories choose to participate in intersectoral and international data exchange and surveillance platforms [5,6,7,8,9]. Although varying in their design, set-up and purpose, these platforms have in common that they perform a joint analysis of heterogeneous multi-user sequence data. As the input of low quality data may negatively impact the joint analysis, users have the responsibility to ensure that all contributed data is quality-controlled. To fully utilize the potential and possibilities provided by intersectoral and international data exchange platforms, a high level of reproducibility, interoperability and therefore common standards are needed [10]. At the same time, redundant analyses, especially of computationally expensive steps, such as assemblies, can be avoided when all results are generated and stored in a structured, exchangeable way. However, when sharing processed data, such as assemblies, it is even more crucial to ensure that prior raw data underwent a stringent quality control. Many factors influence the quality of assemblies. Generally, coverage depth, genome coverage and read accuracy (Q30) are the main factors taken into consideration [2]. Furthermore, sequencing biases, such as GC bias may affect the integrity of the resulting assemblies [11]. In addition, intergenus and intragenus contaminations can have severe effects on downstream analyses [12,13]. Importantly, once sequencing reads have been assembled, it is often impossible to deduct whether anomalies in the assemblies stem from insufficient raw read quality, assembly errors or whether they reflect a biological reality. Indeed, assemblies may appear completely inconspicuous (for example in the case of interspecies contaminations), but are in fact compromised, with the potential to disrupt subsequent analyses [13]. It is therefore mandatory that a stringent quality control, including contaminations checks, is applied before any assembly data is shared.

A number of different bioinformatic pipelines have been developed for the initial analysis of Next Generation Sequencing (NGS) data, each including at least read trimming [14,15] and de novo assemblies as basic steps. Most pipelines implement either SKESA [16] or SPAdes [17], sometimes through the use of unicycler [18] or shovill [19]. Read Quality Control (QC) before and after trimming is also very common, e.g., by applying FastQC [20]. However, only some pipelines additionally perform a subsequent QC on the assemblies with e.g., QUAST [21] (GalaxyTrakr [22], and ARtWORK [23]). Many pipelines run mlst [24] which can be leveraged for contamination detection. SneakerNet [25] and INNUca [26] perform explicit steps to detect contaminations (using duplicated alleles and Kraken taxonomic classification). ASA3P [27] is another pipeline performing a contamination control, which uses fastq screen to detect common contaminants, but does not explicitly search for intergenus or intragenus contaminations [28]). ARtWORK performs a reference search and a subsequent assembly scaffolding. Further analyses not directly related to QC (e.g., serotyping, AMR gene detection, genome annotation, phylogeny) are part of GalaxyTrakr, SneakerNet, ASA3P, nullarbor [29], TORMES [30] and Bactopia [31]. Many of the aforementioned pipelines provide robust results and some have been tested on very large datasets. However, even with the best pipeline in place, decisions on the quality of data have to be made. Here the differing expertise between the developers of analysis pipelines and their final users is especially apparent, as programmers frequently have no insight into species-related peculiarities and biologists may not know how to assess WGS related QC issues. One solution is to provide users with comprehensive and clearly presented information, but to leave final decisions (i.e., approval or discard of the assembly) up to staff with species-specific biological expertise. Up to our knowledge, no existing pipeline provides species-specific guidance for quality assessment.

For this purpose, we have developed AQUAMIS, a Snakemake pipeline for Assembly-based Quality Assessment for Microbial Isolate Sequencing. It performs different quality assessment steps, reference search and assembly as well as taxonomy- and gene-based contamination and completeness detection. Our aim was to design a pipeline for the automated, speedy and structured analysis of large numbers of datasets, which condenses all steps for a rigid QC in one simple command. Importantly, although in principle any species can be analyzed, species-dependent QC thresholds are applied for the species identified by the reference search. The results of the analysis are presented in an user-friendly interactive R Markdown report, which allows a visual assessment of the analyzed data with the help of a color-coded ‘traffic light’ system, utilizing the species-dependent QC thresholds to categorize specific sample parameters as ‘good’, ‘sufficient’ or ‘insufficient’.

With AQUAMIS, we also provide a powerful tool to ensure the compliance with international standards for NGS Data in One Health related settings. For greater interoperability with data from different sources or sectors, all results are additionally made available as structured JSON files, which can readily be exchanged and are machine-readable. This way, the results of different pipelines with comparable scopes can readily be combined into a common, harmonized format to control the quality of data from different providers.

The pipeline is available as open source code on https://gitlab.com/bfr_bioinformatics/AQUAMIS (accessed on 26 April 2021) and easily installable via Bioconda [32] and Docker [33].

For validation, we have tested AQUAMIS on thousands of manually curated isolate sequencing datasets. The pipeline runs robustly and the results provide guidance on proper thresholds that allow a judgement on QC (with a focus on contamination) for different QC metrics. Furthermore, AQUAMIS was utilized to investigate published and hitherto unpublished simulated contamination datasets, in order to compare the best indicators for contamination detection.

## 2. Materials and Methods

### 2.1. Implementation and Availability of AQUAMIS

**Figure 1:**
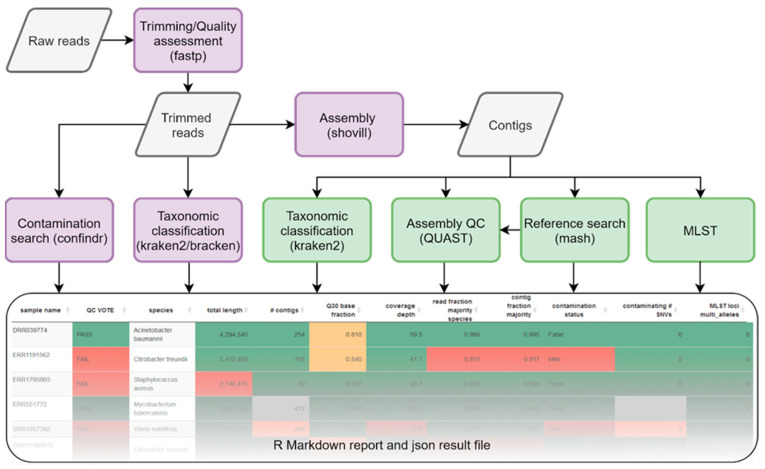
Workflow of AQUAMIS. Raw reads of all datasets are trimmed and quality assessed using fastp. Based on the trimmed reads, contigs are assembled and contaminations are searched, both via a taxonomic classification with abundance estimation and a gene-based approach (purple fields). Based on the assembled contigs, the closest reference to each sample is searched, the assembly quality is assessed, multi-locus sequence typing is performed and contaminations are detected via taxonomic classification again (green fields). The results are presented both in an interactive, configurable R Markdown report and in a structured, computer-readable JSON file. An example report is available at https://bfr_bioinformatics.gitlab.io/AQUAMIS/report_test_data/assembly_report.html, accessed on 26 April 2021.

AQUAMIS is a Snakemake [34] workflow for the user-friendly, structured primary and secondary analysis of bacterial sequencing datasets. It comprises different modules, based on reads and assemblies that together perform a rigid quality assessment of the data (Figure 1). AQUAMIS is called via its python wrapper *aquamis.py* and is provided with a structured sample sheet as input, which can easily be generated using the included script *create_samplesheet.sh*. It is highly configurable using both command line options and a Snakemake config file. For trimming, quality control and quality assessment of raw reads, fastp v 0.20.1 [15] is used. Based on the trimmed reads, a taxonomic classification and abundance estimation is calculated using Kraken2 v 2.1.1 [35] and bracken v 2.5 [36] using the MiniKraken database, or a user-provided database. The results are provided on either a genus or species level and are used to calculate the fraction of reads belonging to the majority species (or genus) in a dataset, giving an estimate of intergenus contaminations. To provide a more accurate measure of intragenus and intergenus contamination detection, ConFindr v 0.7.4 [12] is run via its python API using the provided cgMLST schemes. Additionally, a custom database for *Campylobacter* (see below) and the rMLST-based schemes can be utilized. Raw reads are then assembled using shovill v 1.1.0 [19], exposing all its parameters, in particular the underlying assembler (default: SPAdes) and subsampling of high depth samples (default 100). Based on the assembled contigs, a reference search based on mash v 2.2.2 [37] is executed to determine and download the closest available reference from the NCBI. Users can provide different reference databases and configure the sketch size (default: 1000). AQUAMIS comes with a prepared mash database containing all complete bacterial, plasmid and phage genomes. The use of complete genomes was chosen to provide QUAST with a complete reference genome, which allows a better understanding of the genomic content and the alignment of draft to reference genome. However, this may ultimately lead to a more distant reference genome, as many more draft genomes than reference genomes are available on NCBI. The reason we provided plasmid and phage sequences was to assure that AQUAMIS works also for non-whole-genome (non-bacterial) sequencing projects such as plasmid and phage sequencing. The MLST type is calculated based on the contigs using mlst v 2.19 [24] with the PubMLST database [7]. For an in-depth quality assessment of the assemblies, QUAST v 5.0.2 [21] is run, providing both the contigs and the closest reference sequence. As part of QUAST, the BUSCO [38] module is utilized to estimate both the completeness and possible intergenus and intragenus contaminations. QUAST provides a number of assembly parameters, among them N50, a fraction of recovered reference genes and reference genomes, potential misassemblies and other genes. Its built-in Icarus report aids the visualization of the structure of the draft genome (compared to the complete reference genome) [39]. Additionally, for a more robust taxonomic contamination detection, Kraken2 is run again based on the assembled contigs. This prevents high copy number plasmids from distorting the estimated fraction of the majority species, as these plasmids may be prevalent in different species and therefore assigned incorrectly. Finally, contigs are renamed to their sample name and further metrics are extracted. All results are collected and saved in a structured JSON file, also containing all tool and database versions, as well as the used parameters. A full graph of all steps of the workflow is available in Supplementary Figure S1. All parameters and tool versions are shown in Supplementary Table S3.

Furthermore, AQUAMIS performs an automated QC estimation for all samples. Threshold parameters are chosen based on in-house data and publicly available data from NCBI (Supplementary Data Files SD4 and SD5). AQUAMIS categorizes samples by color according to these criteria and returns a list of failed and passed samples, which can be utilized for downstream analyses. Snakemake manages the execution of all required modules until the desired final output. Module fails (e.g., failure to assemble a sample due to insufficient coverage) are addressed explicitly within AQUAMIS: The workflow keeps track of all failed samples (and modules) and performs a rerun without the failed samples in order to obtain a final report. It also provides the user with the information about each sample’s reason for failure. This strategy allows a smooth, nearly unsupervised assembly of a large number of samples—pointing users to the few exceptional cases for a more detailed fail analysis. Since this so-called business logic cannot be managed automatically by any workflow language, AQUAMIS is to the best of our knowledge the only failsafe workflow in this regard.

The AQUAMIS data structure is designed to maximize reproducibility, machine-readability and to allow easy data sharing. The results of each module, as well as calculations custom to AQUAMIS are saved into separate JSON files for each sample after the two quality control stages, i.e., pre- and post-assembly. This two-step method allows the partial execution of AQUAMIS for sequencing reads-only quality control and still provides contamination information for a sample in case of a genome assembly failure. At the root level of each JSON file, information is split into two branches. The “pipeline” branch stores unaltered results for each module in a full-take approach together with the time of analysis, module version and the command used for execution, where available. The “sample” branch comprises the configuration of AQUAMIS at run-time with paths to the various databases, a summary of selected results from all modules to sufficiently characterize a sample, as well as their assessment according to the AQUAMIS QC thresholds. In addition, we provide the functionality to validate and filter each JSON file, e.g., if sharing of limited information is intended. For this, two permissive JSON schema can be customized by the user to higher stringency. For example, omitting JSON nodes in the filtering schema will prune respective branches from the post-assembly result file and a new, compacted JSON is saved to a filtrate folder. Selected metrics are aggregated from all samples and additionally saved in tabular format to ease post-execution data mining on a per run basis or if JSON parsing is not feasible.

All results are also provided as an interactive HTML report that features different sections to give both a quick overview over the most important results and an in-depth picture of all metrics for quality assessments.

The HTML report is structured in tabs, with each tab dedicated to a different results category. The first tab gives an overview of the analyzed dataset, including a list of ‘failed samples’, i.e., samples for which the execution of a module failed. The second tab lists important QC parameters for each sample, which are color-coded for a better visual assessment. The third tab shows the full detailed results for the read quality assessment, trimming, assembly and contamination detection for all modules. Additional tabs contain in-depth information on trimming, taxonomic classification and contamination analysis. For full reproducibility, the applied program versions and parameters are provided as well. For an example report, see https://bfr_bioinformatics.gitlab.io/AQUAMIS/report_test_data/assembly_report.html, accessed on 26 April 2021.

### 2.2. Datasets and Study Design for Contamination Detection

To demonstrate the ability of AQUAMIS to detect different kinds of contaminations reliably, we analyzed in silico contaminated sequencing reads based on both existing and novel datasets with known ground truth.

#### 2.2.1. Benchmark with In Silico Contamination Data

For validation of the AQUAMIS workflow, all data provided from [40] were downloaded and extracted. The original dataset was designed to study the effect of contaminations on phylogenetic analyses [13]. The dataset consists of artificially mixed sequencing reads from *Listeria monocytogenes*, *Escherichia coli* and *Salmonella enterica* with 248 samples per species. For the present study, each species dataset was analyzed separately with the AQUAMIS workflow, as well as with ConFindr using an rMLST database [12,41]. From these analyses, relevant QC metrics were summarized and contamination predictions were performed using the thresholds from Table 1. AQUAMIS reports for this data are available at https://bfr_bioinformatics.gitlab.io/AQUAMIS_contamination_study/, accessed on 26 April 2021.

#### 2.2.2. Creation of a Novel ConFindr cgMLST Scheme for *Campylobacter* spp.

Since ConFindr does not provide a cgMLST scheme for *Campylobacter* spp., we designed the missing scheme and published it under https://zenodo.org/record/4604758 (accessed on 26 April 2021). The scheme was generated following the ConFindr script create_genus_specific_db.py (https://github.com/OLC-Bioinformatics/ConFindr/tree/master/confindr_src, accessed on 26 April 2021) setting the minimum genome size to 1 Mbp instead of 2 Mbp. In brief, the script downloads all complete Campylobacter spp. genomes (354 complete genomes representing 33 different Campylobacter species) from NCBI RefSeq. It then performs pairwise Nucleotide BLAST with a set of reference genes in order to identify suitable loci for the cgMLST scheme. The reference genes were obtained from the *Campylobacter* reference strain *Campylobacter jejuni* subsp. *jejuni* NCTC 11168 (accession: NC_002163.1). All 1573 genes were renamed and extracted into single files. In contrast to other existing schemes (i.e., for *Salmonella enterica* or *Escherichia coli*), fewer complete genomes were available for the creation of the *Campylobacter* spp. scheme. However, a scheme generated from all 3370 available draft and complete NCBI RefSeq genomes was found to be highly similar. Similarly, we did not observe any major changes when loci from the *Campylobacter* cgMLST scheme from the INNUENDO [8] MLST schema (https://zenodo.org/record/1322564, accessed on 26 April 2021) were provided as reference genes.

#### 2.2.3. Creation of a Contamination Dataset for *Campylobacter* spp.

To complement the previously published contamination datasets from FDA for *L. monocytogenes*, *E. coli* and *S. enterica*, we created a novel simulated dataset for *Campylobacter*. Importantly, compared to *L. monocytogenes*, *E. coli* and *S. enterica*, the genus *Campylobacter* is composed of two predominant (*C. coli* and *C. jejuni*) and several other species. In addition, due to frequent recombinations, hybrid species exist [43]. For the creation of the dataset, we followed the strategy outlined by Pightling et al. [13]. In short, we downloaded all complete *Campylobacter* genomes from RefSeq. Next, we computed the MLST ST using mlst (https://github.com/tseemann/mlst, accessed on 26 April 2021) and excluded all samples without an ST, resulting in a final dataset of 218 samples. We then determined the genetic similarity between these samples by computing pairwise MLST allele distances (using https://github.com/tseemann/cgmlst-dists, accessed on 26 April 2021). For each sample, we attempted to find a close, intermediate and distant matching sample following the proposed definition by Pightling et al.: close (same ST, 0 allele distance (AD)), intermediate (2–6 AD), distant (7 AD). We selected two *C. coli* and six *C. jejuni* samples with at least one close, intermediate and distant matching sample. For each species, we selected genomes with maximal overall genomic diversity and simulated reads from the selected genomes using ART_Illumina v2.5.8 [44] (see Pightling et al. [13] for details). Next, we combined reads from the eight samples and their respective matching samples using the script *select_reads.pl* from http://github.com/apightling/contamination, accessed on 26 April 2021 in order to create simulated contaminated datasets. Additionally, we created intergenus contaminants by mixing reads of the eight *Campylobacter* spp. samples with reads from any of the other three genera (*Listeria*, *Salmonella*, *Escherichia*) of the FDA dataset. The read and assembly data is available at zenodo (https://zenodo.org/record/4601406, accessed on 26 April 2021). A detailed description of the dataset is also available in Supplementary Data Files SD1 and SD2.

### 2.3. Datasets and Study Design for Contamination Detection 

#### 2.3.1. Description of the In-House Dataset

For validation of the AQUAMIS pipeline with in-house data, we analyzed sequencing data of isolates submitted to the German national reference laboratories for Antibiotic resistance, *Salmonella*, *E. coli*, *Listeria monocytogenes* and *Campylobacter* sequenced between 2016 and 2020 for further diagnostic investigations. In total, the dataset encompassed 396 *Campylobacter* spp., 1346 *Escherichia* spp., 2938 *Listeria* spp. and 2722 *Salmonella* spp. isolates. Samples were manually curated to ensure sufficient per base quality, yield, coverage depth and coherence with serotyping and other phenotypic tests. All data were analyzed using AQUAMIS. The assemblies were generated by shovill with SPAdes. Quality metrics were extracted from the AQUAMIS report stats.

#### 2.3.2. Comparison of Taxonomic Classification Based on Reads and Contigs

In order to investigate possible classification biases, we screened the AQUAMIS results obtained for the in-house *Salmonella* isolates, that showed a possible contamination based on the read-based taxonomic classification result (fraction of majority species < 0.90), while showing no contamination based on the contig-based taxonomic classification result (fraction of majority species > 0.95). We detected 58 samples that fulfilled these requirements. For each of these samples, we extracted the following information: Length and coverage depth of each contig, Kraken2 taxonomic classification of each contig (hit1 and hit2 names and abundances) and the plasmid classification according to platon [45].

#### 2.3.3. NCBI Data for Threshold Definition

In order to define thresholds for species not included in our in-house dataset, we downloaded assembly metadata from NCBI available at ftp://ftp.ncbi.nlm.nih.gov/pathogen/Results/, accessed on 26 April 2021. NCBI Pathogen Detection provides assembly metrics for 27 different bacterial genera comprising 70 species. For each of the genera we selected all Illumina-based SKESA assemblies (n ~ 493 k, Supplementary Data File SD5). Based on these, quantile values for total genome length, contig number, and N50 were calculated for all taxa with at least 100 samples. To reflect the intra-genus diversity, the maximum of the upper 95% and the minimum of the lower 5% quantiles among the species of a genus define the genus-wide threshold interval. For genera not available in NCBI Pathogen Detection, we obtained the assembly length from NCBI Genome Reports (n ~ 212 k, Supplementary Data File SD5) available at ftp://ftp.ncbi.nlm.nih.gov/genomes/GENOME_REPORTS/prokaryotes.txt, accessed on 26 April 2021. Latter report also provided the GC ratio for all taxa with at least 100 samples. Again, where different species from one genus were available, genus-wide threshold was derived from species quantiles as described for NCBI Pathogen Detection. In total, we provide QC metrics for 71 genera and 150 species (Supplementary Table S4 and Supplementary Data File SD4).

## 3. Results and Discussion

We have developed the AQUAMIS workflow for the user-friendly, high-throughput primary analysis of microbial isolate sequencing data. The central modules, including trimming, assembly, species identification and contamination analysis are depicted in Figure 1. The pipeline provides users with an interactive HTML report including a fully automated and species-specific preliminary QC decision. 

The following section is structured according to our analysis strategy: Initially, we analyzed >13,000 isolates with AQUAMIS in order to obtain parameter ranges for twelve distinct QC metrics. We then combined these parameter ranges with assembly data from NCBI to define a set of threshold values for the subsequent identification of contaminations on simulated contamination data. Under this controlled setting, the most accurate QC metrics for contamination detection could be derived. Finally, these metrics together with the newly derived threshold values were implemented within AQUAMIS for over 71 genera based on publicly available NCBI data.

### 3.1. Determination of AQUAMIS QC Metrics

**Figure 2:**
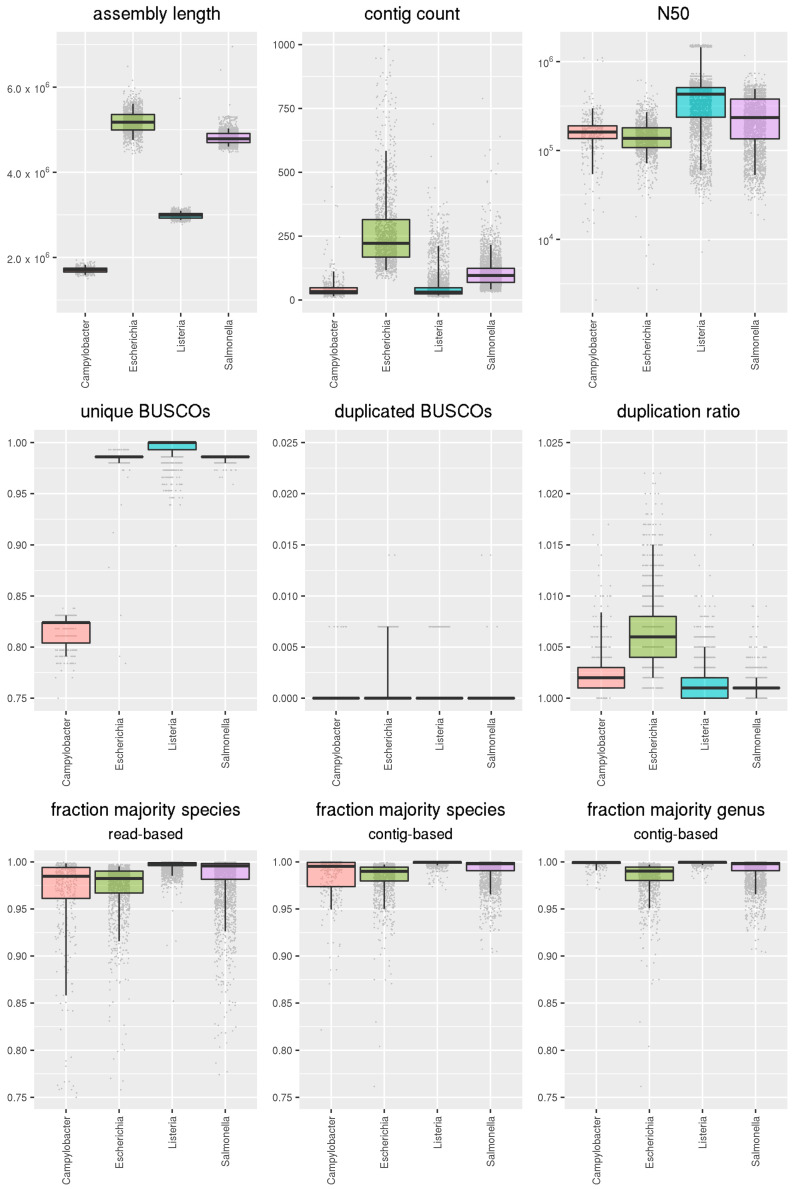
Boxplots of Quality Control metrics for manually curated inhouse data for *Campylobacter, Escherichia, Listeria* and *Salmonella*. The boxes display the median as well as the 25% and 75% quantiles. The lines extend to the 5% and 95% quantiles, respectively. Values outside of the latter are considered outliers and represent potential contaminations.

The AQUAMIS analysis of our in-house dataset (396 *Campylobacter* spp., 1346 *Escherichia* spp., 2938 *Listeria* spp. and 2722 *Salmonella* spp.) shows the ability of the pipeline to be used in high-throughput routine sequencing setups. Based on these results, we show the function of the pipeline and derive species dependent thresholds for various metrics. The results of selected metrics are shown in Figure 2.

Figure 2 shows that the assembly length lies within the expected interval with very low variability for *Campylobacter* and *Listeria*. For *Salmonella* and *E. coli*, we see slightly more variable assembly lengths, which is partially explained by their higher plasmid content [46]. Still, the defined quantiles lie within the thresholds promoted by Timme et al. [2]. The number of contigs is within the expected interval in well-defined ranges, although the variability is again relatively high for *Salmonella* and *E. coli* due to plasmidal contigs. The other assembly-based metric, the N50, behaves congruently. One should note that these two metrics depend more on the underlying assembler than sample related properties. Generally, SKESA [16], which Timme et al. [2] base their values on, is known to produce shorter, less contiguous and more conservative assemblies than SPAdes [17], which we used for our studies.

Besides these basic assembly metrics, we calculated a number of more complex values. For the estimation of both completeness and contaminations, we used BUSCO [38]. The results from our in-house isolate data show that well over 95% of BUSCOs are found for *Escherichia*, *Listeria* and *Salmonella*, indicating a good completeness for these assemblies. For *Campylobacter*, however, we only detected 79–83% of the BUSCOs, indicating that the core genes defined in BUSCO are not present universally in all bacteria. We adjusted the thresholds accordingly. Duplicated BUSCOs, a measure of contamination, are not found in 98% of all samples across all species. More than one duplicated BUSCO was only detected in 0.1% of all samples. We therefore defined a putative contamination with BUSCO as being when more than one single copy orthologue was observed in duplicate. Another measure for possible contaminations is the duplication ratio—the ratio of parts of a reference genome that are covered by two or more contigs from the assembly—can be calculated. For our in-house data, the duplication ratio quantile is highest for *E. coli* with 1.015.

For a more accurate contamination detection, we also show the results from the taxonomic classification of both reads and contigs on the species level, as well as the contigs based on the genus level. The fraction of sequences classified as the majority species are well above 95% in median for all genera. Notably, *Listeria* is close to 100% across all metrics. 

For *Escherichia* and *Salmonella*, we see slightly lower results for the read-based classification which is explained by plasmid content of the isolates, which is often classified as different species and genus. When using taxonomic classification based on the contigs, this effect is largely repealed, as high copy number plasmid sequences appear only once in the assembly. A detailed analysis of this effect is discussed in Section 3.2.4.

For *Campylobacter*, where hybrid species exist and the species boundaries are not as distinct [43], the classification on species level proves difficult. When raised to the genus level, all isolates from our dataset show a fraction of reads assigned to the genus *Campylobacter* near 100%.

#### Thresholds Selected for Contamination Analysis with AQUAMIS

To test the ability of AQUAMIS to predict contaminations, we selected thresholds defining which values are within the expected range for a given genus. The selected values are a composition of thresholds derived from our in-house data, publicly available values from the NCBI and thresholds published by Timme et al. [2].

Table 1 shows the selected thresholds we derived for AQUAMIS in this study. Ranges are defined by the 5% and 95% quantiles of all underlying species. Samples outside of these quantiles are considered as outliers. Q-Score and average coverage depth were adopted from Timme et al. [2]. The assembly length was taken from NCBI Pathogen Detection data (see above) and is in accordance with both Timme et al. [2] and our in-house data. The GC content range was derived from the NCBI data (see above). Thresholds for taxonomic genus/species contamination were adopted from ISO 23418:2020 [42] and confirmed by our in-house results. Threshold ranges for BUSCO and the duplication ratio were not available from NCBI and were defined based on our in-house data. As the values for N50 and number of contigs are highly assembler dependent, these were also defined by our in-house results. ConFindr has a built-in threshold for intergenus and intragenus contaminations (Supplementary Table S1) which we adopted. For MLST, we assumed contamination if at least one allele is duplicated. These definitions were used for the subsequent comparative contamination analysis.

### 3.2. Comparative Analysis of QC Metrics for In Silico Contamination Data Using AQUAMIS

**Figure 3:**
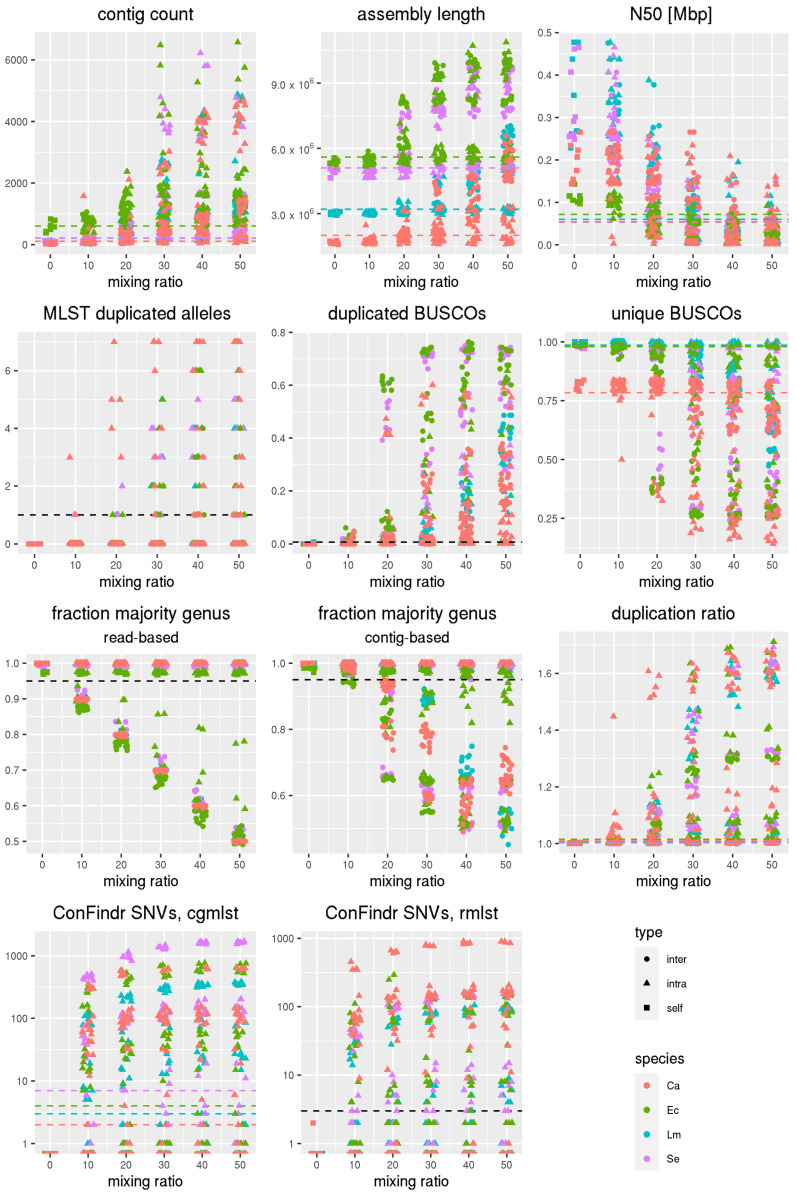
Selected QC results for contamination datasets (for all species): Shown are the respective values according to their mixing ratio. The points are colored by the species and the shape indicates the contamination type (intra, inter, self). The bars show the applied threshold values (black if for all species, colored if species specific).

We ran AQUAMIS on an in silico contaminated dataset published by Pightling et al. [13] supplemented with a novel contamination dataset for *Campylobacter* (see Section 2.2.2). Figure 3 shows the results of the AQUAMIS analysis for selected metrics for different mixing ratios. Dashed lines, colored by the genus they apply for, or in black if they are genus independent, highlight the selected cutoffs. For all shown parameters, the data quality decreases with growing mixing ratios. It is evident that different parameters have a different capability to detect different types of contaminations. While e.g., taxonomic metrics, represented by read-based taxonomic classification and contig-based taxonomic classification have high discriminatory power for intergenus contaminations, metrics like the duplication ratio, MLST, ConFindr, contig count or N50 are more suitable for detecting intragenus contaminations. The exact accuracy of the different parameters for intergenus and intragenus contaminations is discussed in the following sections.

Chosen thresholds are specific, i.e., they almost never show falsely predicted contaminations (Supplementary Figure S2). The only parameter that is not entirely specific is the contig count. For example, high contig counts of more than 800 contigs were observed for some uncontaminated *E. coli* samples. This can be explained by the presence of (difficult to assemble) plasmids in Enterobacteriaceae, such as *E. coli* and *Salmonella*, which significantly affect the contig count. However, increasing the upper bound for the number of contigs is not an appropriate strategy, as there is no natural cutoff. Indeed, the applied threshold is already well above the thresholds proposed by Timme et al. [2].

#### 3.2.1. Detection of Intergenus Contaminations

Selected results of the AQUAMIS analysis of the in silico contaminated datasets are shown in Table 2 and Supplementary Figure S2. Overall, results show that intergenus predictions are more sensitive for higher mixing ratios across all metrics. The best prediction for intergenus contaminations in all mixing ratios (not itemized in table, see Supplementary Figure S3) can be obtained from read-based taxonomic classification, giving a perfect result. ConFindr based on both cgMLST and rMLST gives near-perfect results as well. However, in some cases ConFindr erroneously predicted an intergenus contamination for intragenus contaminated samples, e.g., a significant *Citrobacter* contamination in two *E. coli* datasets. Thus, ConFindr is less specific than Kraken2. Less accurate, but still applicable are results from the following metrics: contig count, duplicated BUSCOs, unique BUSCOs, contig-based taxonomic classification and assembly length. For these, the sensitivity varies with the species and/or mixing ratio, with poor performances for 10% contamination rates, acceptable performances at a 20% contamination ratio and near-perfect predictions at a 30% contamination ratio (Supplementary Figure S3). The other metrics, namely the duplication ratio, MLST, GC content and N50 show little to no visible effect when presented with intergenus contaminated data. Obviously, the GC content only distinguishes between species with significantly different GC contents and sufficiently large mixing ratios. For MLST, no comparable results were obtained for different species as different schemas consisting of different loci were used.

#### 3.2.2. Detection of Intragenus Contaminations

Regarding intragenus contaminations, the sensitivity of all metrics is largely influenced by the genomic distance of the contaminant. We therefore show the results of the selected metrics for distant (0.05%), intermediate (0.5%) and closely related (5%) contaminants (Table 3). Closely related contaminants (e.g., with zero 7-gene allele distance) are difficult to predict for all metrics. The mixing ratio still has an effect on the sensitivity of contamination predictions, but the effect is much smaller than for interspecies contaminations, affecting mixing ratios of 10% for some metrics (Supplementary Figure S4). 

The best predictors produced by AQUAMIS for intragenus contaminations are the results of ConFindr based on cgMLST, followed by ConFindr based on rMLST. The former increases the sensitivity significantly (see Section 3.2.3). We therefore decided to provide an additional cgMLST scheme for ConFindr for *Campylobacter*.

Following a comparable approach, the metrics BUSCO (unique and duplicated) and MLST were calculated. While BUSCO still works relatively well for distant genomes and has a relatively high signal for close genomes, MLST only has predictive power for distant genomes, which is expected given that only seven highly conserved genes are considered.

Contig count, assembly length and duplication ratio show relative good prediction performance even for intermediate and close genomes. The N50 behaves comparable to the contig count, but still allows less accurate predictions. Taxonomic classification on reads and contigs as well as the GC content are unqualified metrics to detect intragenus contaminations. 

It is notable that for closely related contaminants, various metrics (contigs, duplication ratio, N50) have higher predictive power than the overall best metric, ConFindr. This confirms that a combination of metrics can enhance the ability to predict contaminations, giving the combined modules of AQUAMIS a real advantage over the single tools.

#### 3.2.3. Comparison of ConFindr Results Using the cgMLST and rMLST Database

The present analysis allows an in-depth comparison of the sensitivity of two different approaches for the detection of contaminations using ConFindr. We compared the validated approach of ConFindr in combination with an rMLST scheme [12], against ConFindr in combination with a cgMLST scheme. Overall, we could show that cgMLST is more sensitive than rMLST—throughout all genera and all distances (61% to 73% sensitivity and same specificity) (compare Section 3.2.2). This was confirmed by comparing the number of single-nucleotide variants (SNVs) for each method (Figure 3). For *Listeria*, *E. coli* and *Salmonella* the number of SNVs was found to be much greater for cgMLST than for rMLST. In order to detect contaminations, a greater number of SNVs is preferable, as this allows the identification of closely related contaminants, or of contaminants present at low levels. As the number of possible SNVs correlates directly to the size of the scheme, a greater number of SNVs with a larger cgMLST scheme is to be expected (Supplementary Figure S5). Indeed, we found that for *Campylobacter*, the number of SNVs is similar for cgMLST and rMLST, as the number of considered loci and bases of the newly developed *Campylobacter* cgMLST scheme is of similar size as the rMLST scheme (see Supplementary Table S1). ConFindr also attempts the prediction of the contamination ratio. Both methods perform similarly with a mean deviation of 7.4 (cgMLST) and 7.9 (rMLST) between the predicted and simulated contamination mixing ratio.

Overall, we found that ConFindr achieves better contamination predictions when supplied with a cgMLST scheme, compared to an rMLST scheme, as cgMLST schemes are generally larger and thus provide a greater number of potential SNVs, which can be used for differentiation. However, only a limited number of cgMLST schemes is available to date and thus rMLST remains a very useful alternative. Since the rMLST scheme underlies copyright restrictions, the creation of more genus specific cgMLST schemes should be endeavored by the research community.

#### 3.2.4. Taxonomic Classification Analysis for Small, Highly Abundant Plasmids

The previous analysis on the simulated contamination data revealed that read-based taxonomic classification is more sensitive than contig-based taxonomic classification. In particular, for mixing ratios of 10–20%, contig-based taxonomic classification underperforms, whereas above the 20% mixing ratio, the accuracy for both methods is 1. This is because for small mixing ratios, reads from the contaminant are either not part of the assembly or are included into contigs of the subject. In the latter case, the subject species dominates the taxonomic classification since only the best hit per contig is accounted for. For larger mixing ratios, reads originating from contaminant species assemble into separate contigs.

However, the information from contig-based taxonomic classification provides complementary information for species containing multi copy plasmids (which occur frequently in Enterobacteriaceae) [46]. This is due to two reasons: Firstly, plasmids may not be correctly taxonomically classified to an organism, as similar plasmids may be associated to other taxa, leading to potential misclassification of plasmid sequences to a different taxon. Secondly, reads from multi-copy plasmids occur with increased frequency compared to reads of the chromosome. Thus, their contribution to the overall assembly length is strongly overestimated in the entire genome. For these reasons, read-based taxonomic classification may be unspecific in the presence of small multi-copy plasmids and yield false contamination predictions. Conversely, contig-based taxonomic classification normalizes the effect and thus reduces multi-copy and misclassification bias. In order to explore this further, we collected 58 samples from the in-house *Salmonella* strain collection where read-based taxonomic classification indicates an intergenus contamination (fraction of majority genus < 90%) and contig-based taxonomic classification does not (fraction of majority genus > 95%). The median difference in classification was 11%.

**Figure 4:**
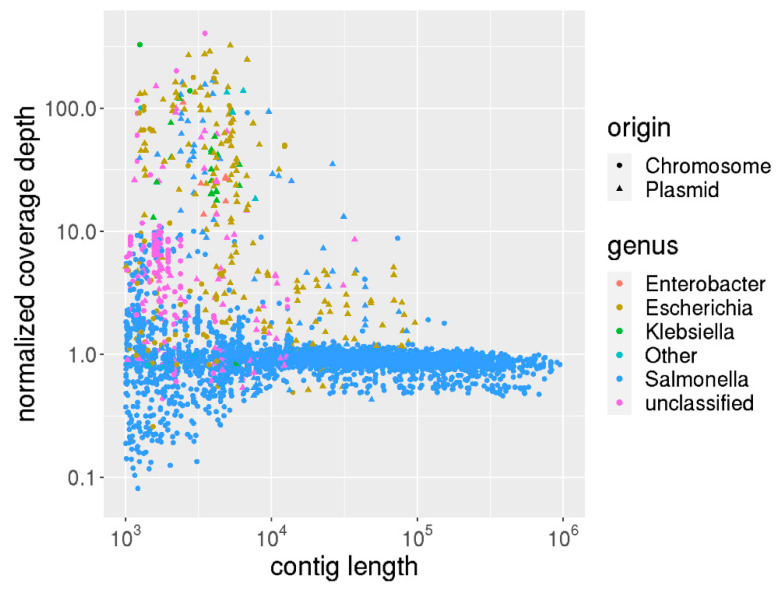
Taxonomic classification based on reads and contigs comparison. Compared is the normalized coverage depth vs. the contig length of all contigs from a set of 2721 *Salmonella enterica* that show different taxonomic classifications based on reads and contigs. Circles denote contigs predicted as chromosomal origin and triangles from plasmids. The coloring indicates the genus from the taxonomic classification of that contig. Clearly, short contigs originating from plasmids frequently occur in high-copy number and are associated to other genera than *Salmonella*.

Figure 4 describes the contig lengths, their respective coverage depths as well as their taxonomic and plasmid classification. While the chromosomal contigs have uniform depth, the contigs associated with plasmids frequently display an increased depth (10–100 fold) and are associated with other Enterobacteriaceae. This explains the discrepancy of read- and contig-based taxonomic classification, as the short multi-copy plasmids may be associated to different plasmids and are counted disproportionately to their genomic contribution when counting reads. Thus, we could show that the bias from multi-copy plasmids can be reduced (or avoided altogether) when considering contig-based taxonomic classification. Therefore, for a more comprehensive analysis the consultation of both metrics—read- and contig-based taxonomic classification—is advisable.

#### 3.2.5. Discussion of QC Metrics for Contamination Detection

The role of the different QC metrics demonstrated above in detail shows different predictive power for the detection of contamination types. A general overview over all measures is given in Supplementary Table S2. Table 2 and Table 3 summarize the predictions for intergenus and intragenus contaminations. ConFindr cgMLST performs best overall, closely followed by ConFindr rMLST. Thus, when a cgMLST scheme is available, ConFindr cgMLST is preferred and when only the rMLST scheme is available, it is the recommended method of choice.

However, for intergenus contaminations, taxonomic classification based on reads achieves the highest sensitivity. This also has the advantage of a more straightforward interpretation, namely the fraction of reads assigned to different species or genus. This is in agreement with the advice of the ConFindr authors to use metagenomics tools to detect intergenus contaminations accounting for less than 5% of the reads of a sample [47] reliably. Although ConFindr excels all other methods for intragenus contaminations, it fails to accurately predict contaminations with closely related contaminant strains of the same species. A number of other methods are more accurate here, i.e., contig count, unique/duplicate BUSCOs, assembly length and duplication ratio. Hence, it becomes clear that the simultaneous inspection of different QC metrics—as available in the AQUAMIS report—provides the best possible information to assess potential intergenus and intragenus contaminations.

### 3.3. Thresholds for Automated QC Decision in AQUAMIS

As a result from the previous contamination analysis, we implemented the following automated and species-specific QC decisions. (i) Pass: Samples are considered as passing QC, when the values for Q30 base fraction, coverage depth, assembly length, the combination of both Kraken classifications and the ConFindr contamination status are within the predefined threshold values. (ii) Warning: If the above requirements are fulfilled, but one value exceeds a warning threshold, a warning is issued. Similarly, if values for the number of contigs, N50, GC content, MLST allele duplication, unique BUSCOs, duplicated BUSCOs or duplication ratio are outside the predefined threshold values, a warning is returned. (iii) Fail: Samples are considered as failing QC when at least one value for Q30 base fraction, coverage depth, assembly length, the combination of both Kraken classifications or the ConFindr contamination status are outside the predefined threshold values.

As described in the previous section, the threshold values were inferred from the combination of NCBI and in-house data. Though initially thresholds were only available for the four genera studied, we were able to extend the automatic QC to a much larger set of genera by including NCBI data. Where NCBI data contained information for assembly length, number of contigs, N50 and GC content, these were utilized to derive species or genus specific thresholds. Other thresholds important for the QC decision are species-agnostic.

Thus, a sketch for a QC decision is available for more than 70 genera (Supplementary Table S4 and Supplementary Data File SD4). If for a given taxon no QC thresholds are available, these values are not considered for the QC decision. All QC decisions are summarized in the AQUAMIS report and color coded in green (pass), orange (warning) and red (fail).

## 4. Conclusions

With AQUAMIS, we provide a robust, easy to deploy pipeline for the routine primary analysis of whole genome bacterial isolate sequences. The pipeline is designed to be very easy to use, both in execution, as well as in the result interpretation. Importantly, while the tool execution requires no prior knowledge on the samples to be analyzed, the results include meaningful, species-specific recommendations guiding the quality assessment of each sample.

By defining species-specific cut-off values for each parameter in accordance with the ISO recommendations, users can automate the sample acceptance or rejection process. This provides a great opportunity for standardization and harmonization and ensures that generated WGS data consistently provide sufficient quality for downstream analysis. This is especially useful for laboratories with high sample throughput, where an individual sample check is not feasible. Nevertheless, a more in-depth, manual and individualized evaluation, especially of rejected samples, is recommended to avoid unnecessary and expensive redundant sequencing. Especially for rare strains, novel strains, hybrid strains or strains with high plasmid content, the standard cut-off may be too conservative.

A novel approach implemented by AQUAMIS is the contig-based taxonomic classification. This strategy allows a more accurate determination of the species, unbiased by the presence of high-copy-number plasmids and thus provides the basis for a better estimation of actual contaminations. For contamination detection, we implemented the ConFindr tool, for which we designed a new cgMLST scheme in order to extend its usability towards the genus *Campylobacter*. We also performed an independent benchmarking of the overall performance of the novel cgMLST functionality of ConFindr. Our results clearly show that the novel cgMLST-based approach is preferable to the classic rMLST-based approach for all tested genera. The final results generated by AQUAMIS are presented in a user-friendly, configurable and interactive R Markdown report for on-site QC, as well as highly structured, computer readable JSON reports, which ensure interoperability for subsequent data sharing. Through the use of Snakemake and additional wrappers to catch business logic errors, the pipeline is extremely failsafe and will resume automatically where failures of individual modules, samples or manual interaction have disrupted the workflow. At the same time, it is highly configurable via both command line options and config-files, exposing most major parameters of the underlying tools. 

AQUAMIS is available as open source code from https://gitlab.com/bfr_bioinformatics/AQUAMIS, accessed on 26 April 2021 and installable via Docker and Bioconda. Through its open design, it is available to anyone for on-site installation, circumventing data privacy problems and restricted access and availability of exclusively cloud based approaches. As the pipeline is based on Snakemake, it is also possible to run AQUAMIS in cluster and cloud environments (untested to date). 

In this study, we furthermore present a comprehensive analysis of typical assembly and QC based parameters for different microbial genera. Together with other similar studies, our data collection and subsequent analyses contribute to the ongoing discussion on standardization of sequencing data in a One Health context to ensure compatibility between data from different sources and sectors. 

## Figures and Tables

**Table 1:** Quality Control thresholds for isolate sequencing data.

Quality Metric.	*Salmonella enterica*	*Listeria monocytogenes*	*Escherichia coli*	*Campylobacter* spp.
Q-score	≥30 ^a^			
Average coverage depth	≥30 ^a^	≥20 ^a^	≥40 ^a^	≥20 ^a^
Assembly length [Mbp]	4.5–5.1 ^e^	2.8–3.2 ^e^	4.5–5.6 ^e^	1.4–2.0 ^e^
Number of contigs	42–216 ^b^	18–212 ^b^	116–618 ^b^	15–112 ^b^
Species purity [%]	>95 ^c,d^			
GC content [%]	48.1–56.1 ^e^	33.9–41.9 ^e^	42.6–54.6 ^e^	26.4–35.3 ^e^
Unique BUSCOs [%]	>95 ^c^			>79 ^b^
Duplicate BUSCOs	≤1 gene ^c^			
Min N50 [kbp]	53.0 ^b^	60.0 ^b^	71.7 ^b^	54.2 ^b^
Max Duplication ratio	1.002 ^b^	1.005 ^b^	1.015 ^b^	1.009 ^b^

Thresholds reflect data from different sources. (**a**): as described by Timme et al. [2]; (**b**): observed on in-house data, 5% and 95% quantiles of values; (**c**): based on in-house observation, manually chosen; (**d**): described in ISO norm 23418:2020 [42], (**e**): 5% and 95% quantile observed on publically available data from NCBI.

**Table 2:** Sensitivity of intergenus predictions for various QC metrics by species (averaged over mixing ratio and contaminant species). Values are the number of contamination predictions (QC metrics above or below thresholds) [TP] divided by number of contaminated samples [P]. Good predictions (>0.8 accuracy) are colored in light blue, sufficient predictions (>0.2) in orange and insufficient predictions in white. Perfect predictions are shown in bold.

Predictor	*Campylobacter* spp.	*E. coli*	*L. monocytogenes*	*S. enterica*
ConFindr cgMLST	0.98	**1.00**	**1.00**	**1.00**
ConFindr rMLST	**1.00**	**1.00**	0.99	**1.00**
# contigs	0.79	0.90	0.60	0.88
Dupl. BUSCO	0.55	0.89	0.56	0.82
Duplication ratio	0.00	0.40	0.00	0.41
GC content	0.35	0.00	0.35	0.40
Kraken2 contigs	0.79	0.89	0.60	0.80
Kraken2 reads	**1.00**	**1.00**	**1.00**	**1.00**
Duplicated mlst	0.00	0.21	0.00	0.11
N50	0.38	0.76	0.40	0.42
Unique BUSCOs	0.41	0.82	0.42	0.75
assembly length	0.64	0.85	0.59	0.80

**Table 3:** Prediction sensitivity for intragenus contamination for different QC metrics and genomic distance of contaminants (averaged over mixing ratio and species). Values are the number of contamination predictions (QC metrics above or below thresholds) [TP] divided by number of contaminated samples [P]. Good predictions (>0.8 accuracy) are colored in light blue, sufficient predictions (>0.2) in orange and insufficient predictions in white. Very good predictions (>0.99) are shown in bold.

Predictor	Distant	Intermediate	Close
ConFindr cgMLST	**1.00**	**0.994**	0.206
ConFindr rMLST	**1.00**	0.713	0.131
# contigs	0.87	0.838	0.438
Dupl. BUSCO	0.79	0.200	0.019
Duplication ratio	0.94	0.787	0.269
GC content	0.00	0.000	0.000
Kraken2 contigs	0.20	0.094	0.013
Kraken2 reads	0.25	0.000	0.000
Duplicated mlst	0.64	0.094	0.000
N50	0.70	0.769	0.237
Unique BUSCOs	0.72	0.619	0.044
assembly length	0.80	0.450	0.119

## Data Availability

All code is open source and freely available at https://gitlab.com/bfr_bioinformatics/AQUAMIS, accessed on 26 April 2021. Installable packages are available Bioconda and Docker. An example report is available at https://bfr_bioinformatics.gitlab.io/AQUAMIS/report_test_data/assembly_report.html, accessed on 26 April 2021. Reports of the contamination study data are available at https://gitlab.com/bfr_bioinformatics/AQUAMIS_contamination_study, accessed on 26 April 2021. The individual predictions of each sample is also available in Supplementary Data File SD3. The cgMLST scheme for Campylobacter spp. is available at https://zenodo.org/record/4604758, accessed on 26 April 2021. Read and assembly data for the Campylobacter contamination dataset is available at https://zenodo.org/record/4601406, accessed on 26 April 2021 and a detailed description is also available in Supplementary Data Files SD1 and SD2. All derived thresholds are available in Supplementary Data Files SD4 & SD5.

## Supplementary Materials

The following are available online at https://www.mdpi.com/article/10.3390/genes12050644/s1, Figure S1: Directed acyclic graph (DAG) of all rules in AQUAMIS, Figure S2: Specificity of different QC metrics for self data without contamination, Figure S3: Sensitivity of intergenus contamination detection, Figure S4: Sensitivity of intergenus contamination detection, Figure S5: Comparison of confindr intra-genus predictions for rMLST and cgMLST, Table S1: Confindr summary for cgMLST and rMLST intra-species analysis, Table S2: Summary of QC metrics, Table S3: Software versions and default parameters for all modules in AQUAMIS, Table S4: Genera and Species for which AQUAMIS provides QC thresholds. Supplementary Data Files SD1 and SD2 provide details about the Campylobacter dataset, SD3 lists all predictions for all samples in the contamination dataset, SD4 provides all derived thresholds, SD5 summarizes the NCBI datasets used for threshold derivation.
