# Supplementary material for: Species-Specific Quality Control, Assembly and Contamination Detection in Microbial Isolate Sequences with AQUAMIS
Source: Genes (Basel). 2021 Apr 26;12(5):644. doi: 10.3390/genes12050644 (PMC8145556; doi:10.3390/genes12050644)
Supplement: Supplementary file 1 [file genes-12-00644-s001.zip › AQUAMIS Supplementary materials.pdf]

AQUAMIS, a Snakemake workflow for assembly and quality assessment of microbial sequences

Carlus Deneke[1], Holger Brendebach[1], Laura Uelze[1], Maria Borowiak[1], Burkhard Malorny[1], Simon H. Tausch[1*]

[1]Department Biological Safety, German Federal Institute for Risk Assessment, Berlin, Germany;* Correspondence: simon.tausch@bfr.bund.de

Supporting Data

The following files are contained in the file supporting_data.zip:

- SD1_genome_info_Ca.tsv: Table with information about subject and contaminant genome accessions as well as MLST ST
- SD2_metadata_Ca.tsv: Table with details about in-silico mixing of Campylobacter contamination dataset
- SD3_predictions_contaminationdata.tsv: Table with all AQUAMIS results and predictions for all samples in all contamination datasets
- SD4_AQUAMIS_thresholds.xlsx: AQUAMIS QC threshold definitions for all defined species and genera in tabular format. In the AQUAMIS application, values are queried from the Gitlab repository file “AQUAMIS/resources/AQUAMIS_thresholds.json”. Supplementary Table S4 provides a list of available taxons.
- SD5_NCBI_dataset_summary.xlsx: Summary of the NCBI datasets from NCBI Pathogen Detection and NCBI Genome Reports. Only species and genera with at least 100 samples were considered for statistical analysis.

Supplementary figures

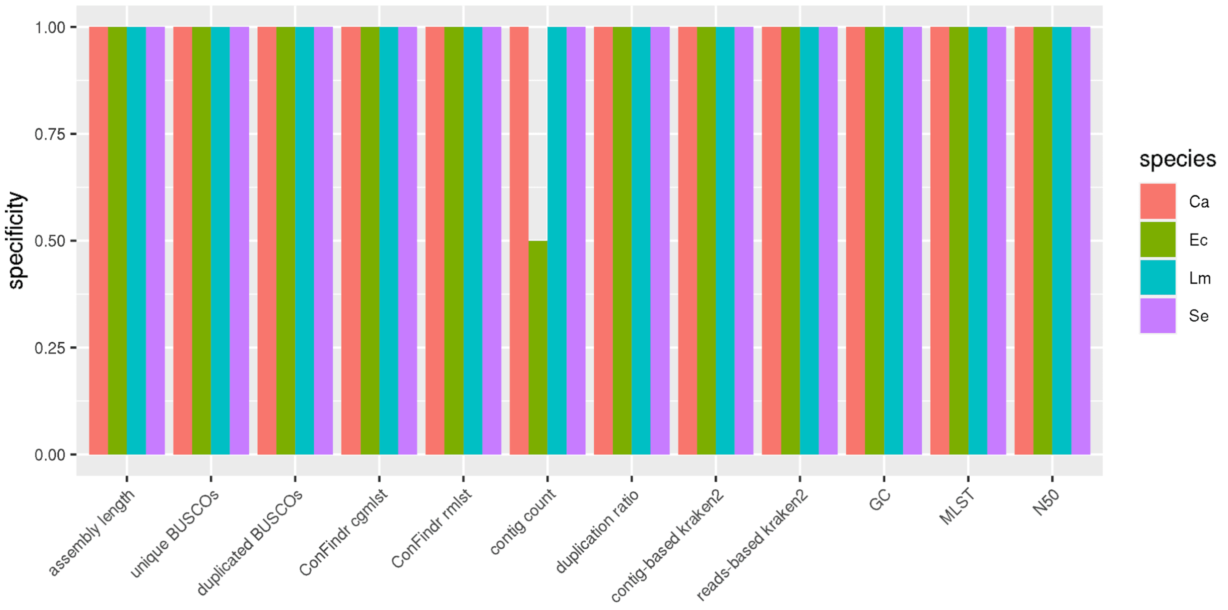

Supplementary FIGURE 2: Specificity of different QC metrics for *self* data without contamination: The AQUAMIS analysis reveals that QC metrics achieve a perfect specificity indicating no contamination for the self samples for all genera studies. Notably, the contig count for E.coli surpasses the the selected threshold in some cases - likely due to the presence of plasmids.

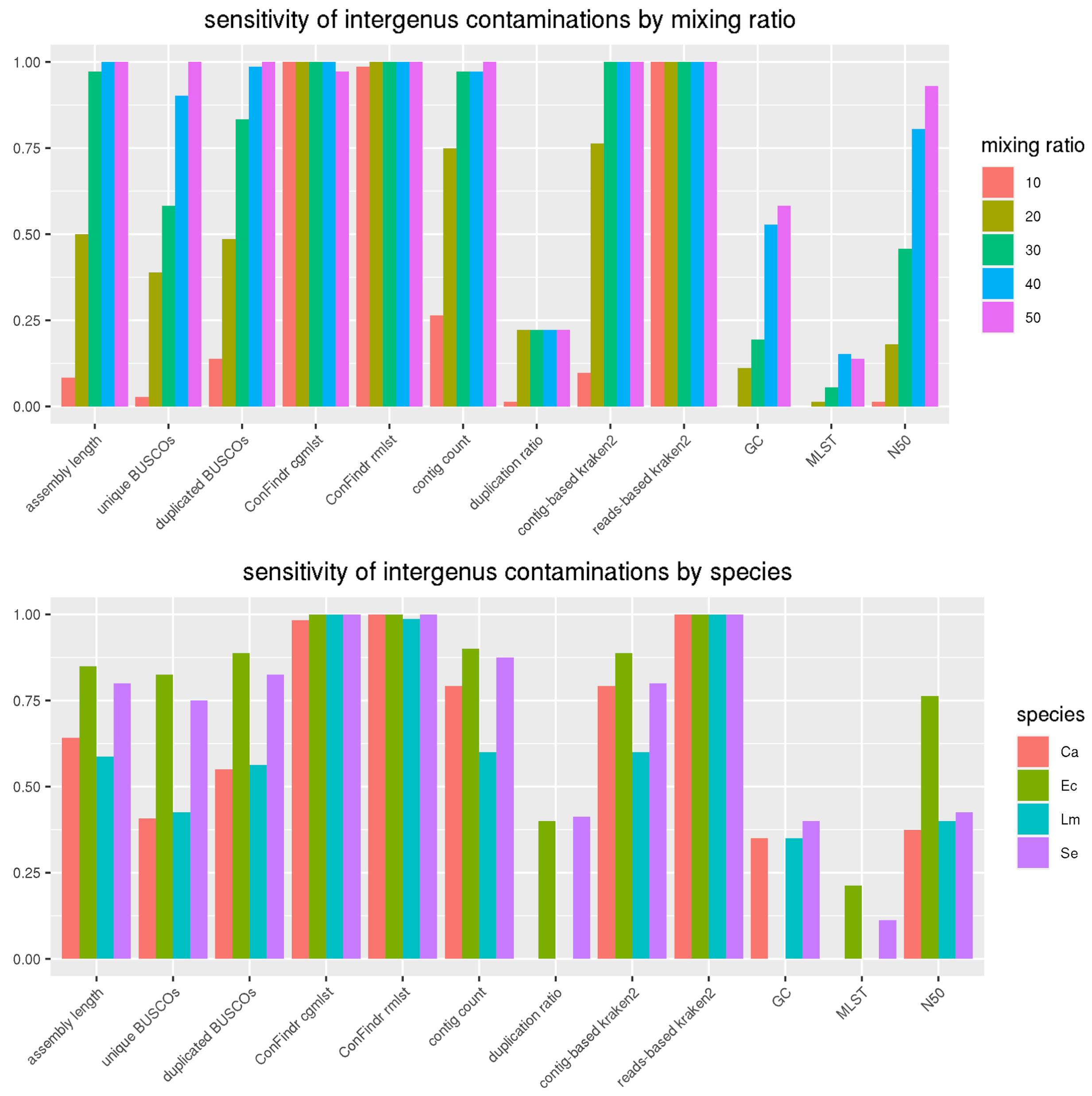

Supplementary FIGURE 3: Sensitivity of intergenus contamination detection: Shown is the sensitivity according to AQUAMIS for all intergenus contamination datasets. Top: Divided by mixing ratio, Bottom: Divided by subject genus.

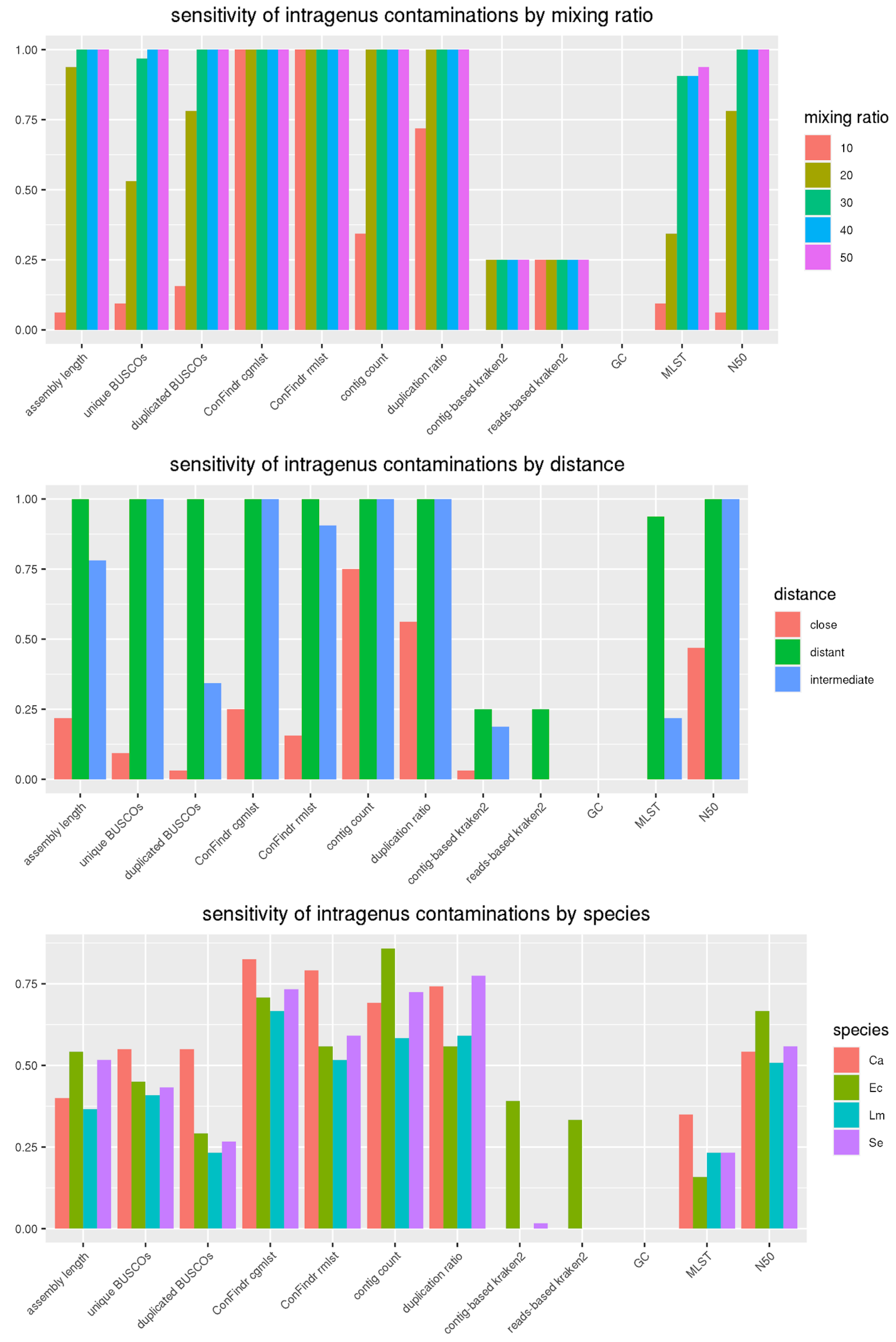

Supplementary FIGURE 4: Sensitivity of intergenus contamination detection: Shown is the sensitivity according to AQUAMIS for all intragenus contamination datasets.Top: By mixing ratio, Middle: By genetic distance, Bottom: By species.

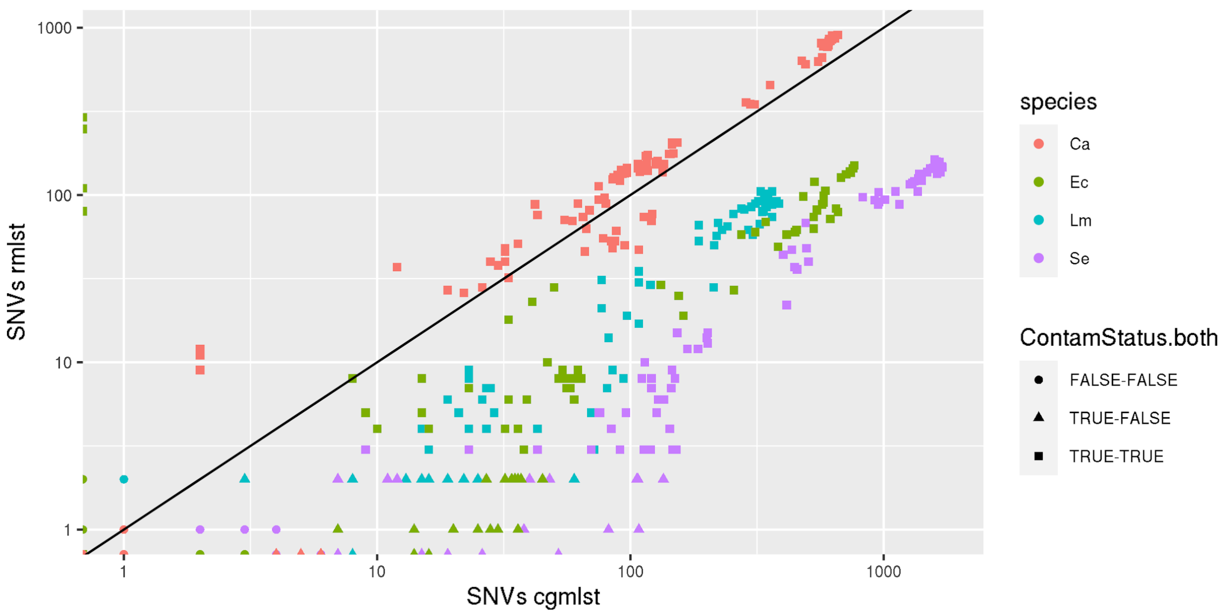

Supplementary FIGURE S5: Comparison of confindr intra-genus predictions for rMLST and cgMLST (logscale). Shown is the number of detected SNVs when applying the genus specific cgMLST scheme (x-axis) or the rMLST scheme (y-axis). The dots are colored by species and the shape denotes the predicted contamination status. (False-False means predicted as False by both methods, True-False predicted by cgmlst as contaminated and by rmlst as non-contaminated, etc.). For the established schemes for Lm, Se, Ec the number of SNVs is substantially higher for cgMLST than for rMLST. Conversely, for Ca it is similar. The Ec samples with zero cgMLST SNVs (far left) are samples that were erroneously predicted as inter-species contaminated (E.coli:Citrobacter).

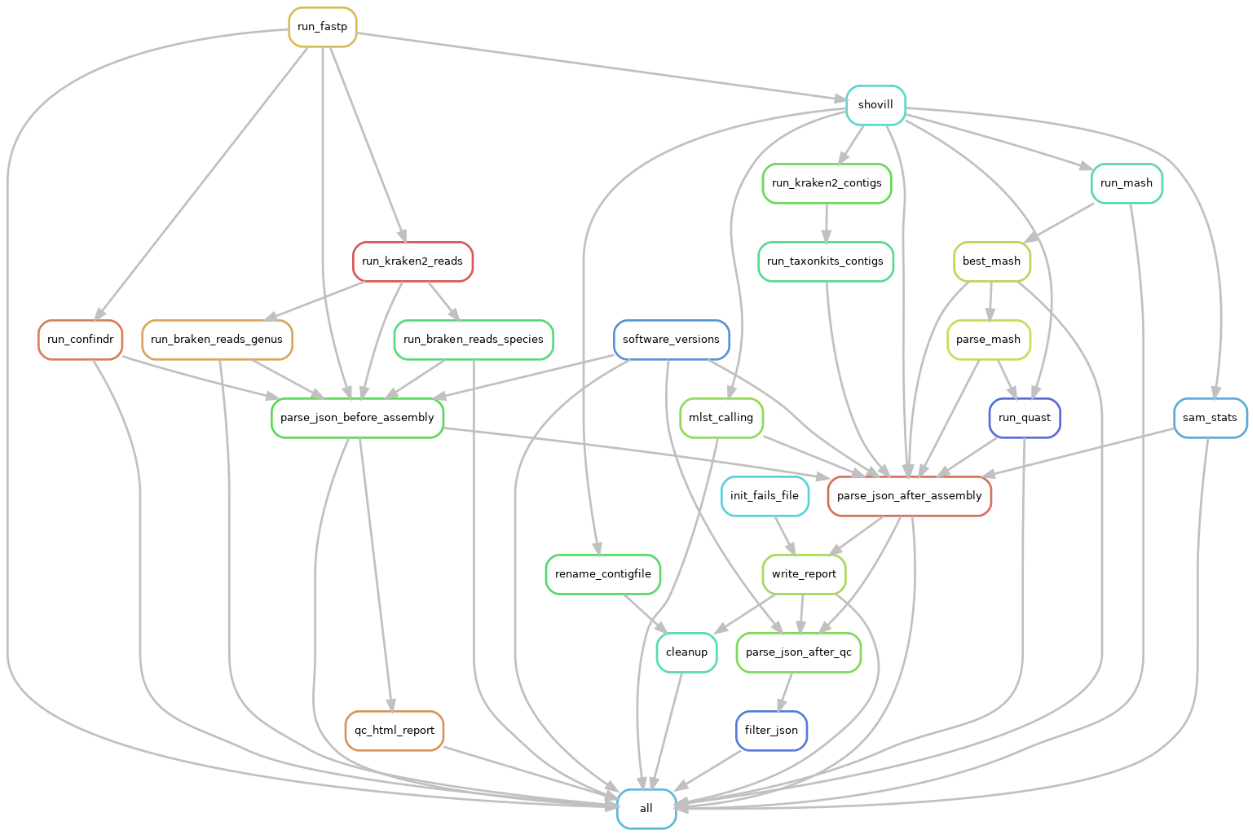

Supplementary FIGURE S1: Directed acyclic graph (DAG) of all rules in AQUAMIS.

Supplementary tables

Supplementary Table S1: Confindr summary for cgMLST and rMLST intra-species analysis. (* by definition)

	Lm	Se	Ec	Ca
cgMLST				
bases_examined.cgmlst	28425	61956	38310	17385
loci.cgmlst	47	116	58	40
SNV_threshold.cgmlst	**3**	**7**	**4**	**2**
minSNV_TP.cgmlst	3	7	7	2
maxSNV_FN.cgmlst	2	6	3	1
uniqueTP.cgmlst	18	17	18	4
rMLST				
bases_examined.rmlst	20181	20862	20940	20691
loci.rmlst	51	51	51	52
SNV_threshold.rmlst*	3*	3*	3*	3*
minSNV_TP.rmlst	3	3	0	9
maxSNV_FN.rmlst	2	2	2	1
uniqueTP.rmlst	0	0	0	0

Supplementary Table S2: Summary of QC metrics.

predictor	Description	Evaluation	Rating inter	Rating intra
confindr.cgmlst	Mash and SNP analysis	Works well for intra and inter species (except close contaminations)	+++	+++
confindr.rmlst	Mash and SNP analysis	Works well for intra and inter species (except close contaminations)	+++	+++
contigs	Atypically high number of contigs (assembly is more fragmented)	Does not work well for inter in Ca and Lm; has strong signal for close intra	+	++
duplicated.orthologs	One or more duplicated single copy orthologs	Works well for distant intra	+	+
Duplication ratio	Untypical number of duplication events	Only for intra contamination; signal for closely related contaminants	-	+
GC	Deviation from a species' typical GC	Works only when inter species contamination combines pairs with different GC	+	-
kraken2_contigs	Fraction of assembled genome is assigned to other species/genus	Sound performance for inter; no signal for intra	+	-
kraken2_reads	Fraction of reads is assigned to other species/genus	Perfect for inter; no signal for intra	+++	-

mlst	One or more allele is found more then once	Only signal for intra and when contaminant is not too close (has different ST)	-	+
N50	Atypically low value for N50 (assembly is more fragmented)	Similar (but weaker) to contig count	(+)	+
single.copy.orthologs	Not all single-copy orthologe are found (genome is incomplete)	Good signal for inter and even better for intra. Requires mixing ratio > 30 %	+	++
total.length	Total length of assembly is atypically long (or short)	Signal for inter and intra but never perfect/ Perfect only for mixing ratios > 30%	+	+

Supplementary Table S3: Software versions and default parameters for all modules in AQUAMIS.

**Module**	Software	Version/ Parameters
**Trimming**	fastp	0.20.1<br>--detect_adapter_for_pe --n_base_limit 50<br>--length_required 15
Taxonomic classification	kraken2	2.1.1<br>--gzip-compressed --paired
	bracken	2.5<br>-r 150 -l G -t 0
	taxonkit	0.7.2<br>reformat --miss-rank-repl "unclassified"
Contamination detection	ConFindr	0.7.4<br>forward_id='_R1', quality_cutoff=20, base_cutoff=2, base_fraction_cutoff=0.05, data_type='Illumina', cross_details=True
**Assembly**	shovill	1.1.0<br>--noreadcorr --assembler spades --depth 100
	spades	3.14.1<br>--isolate
Reference identification	mash	2.2.2<br>-k 21 -s 1000
Assembly QC	QUAST	5.0.2<br>--conserved-genes-finding --reference --features<br>--min-identity 80
MLST	mlst	2.19.0

Supplementary Table S4: Genera and Species for which AQUAMIS provides QC thresholds (see SD4_AQUAMIS_thresholds.xlsx).

**Genus**	Acinetobacter, Aeromonas, Akkermansia, Bacillus, Bacteroides, Bifidobacterium, Bordetella, Brucella, Burkholderia, Campylobacter, Chlamydia, Citrobacter, Clostridioides, Clostridium, Corynebacterium, Cronobacter, Cutibacterium, Enterobacter, Enterococcus, Erwinia, Escherichia, Eubacterium, Faecalibacterium, Flavobacterium, Francisella, Gardnerella, Haemophilus, Helicobacter, Klebsiella, Lacticaseibacillus, Lactiplantibacillus, Lactobacillus, Lactococcus, Legionella, Leptospira, Ligilactobacillus, Limosilactobacillus, Listeria, Mannheimia, Microcystis, Moraxella, Mycobacterium, Mycobacteroides, Mycoplasma, Neisseria, Oenococcus, Parabacteroides, Parcubacteria, Pasteurella, Patescibacteria, Phocaeicola, Prevotella, Priestia, Proteus, Pseudomonas, Ralstonia, Rhizobium, Salmonella, SAR202, Serratia, Shigella, Sinorhizobium, Staphylococcus, Stenotrophomonas, Streptococcus, Thaumarchaeota, Thermoplasmata, Vibrio, Xanthomonas, Xylella, Yersinia
**Species**	Acinetobacter baumannii, Acinetobacter nosocomialis, Acinetobacter pittii, Aeromonas veronii, Akkermansia muciniphila, Bacillus anthracis, Bacillus cereus, Bacillus licheniformis, Bacillus pumilus, Bacillus subtilis, Bacillus thuringiensis, Bacillus toyonensis, Bacillus velezensis, Bacillus wiedmannii, Bacteroides fragilis, Bacteroides uniformis, Bifidobacterium bifidum, Bifidobacterium breve, Bifidobacterium longum, Bordetella pertussis, Brucella abortus, Brucella melitensis, Burkholderia cenocepacia, Burkholderia cepacia, Burkholderia gladioli, Burkholderia multivorans, Burkholderia pseudomallei, Burkholderia ubonensis, Chlamydia trachomatis, Citrobacter freundii, Clostridioides difficile, Clostridium beijerinckii, Clostridium botulinum, Clostridium perfringens, Clostridium sporogenes, Corynebacterium diphtheriae, Corynebacterium pseudotuberculosis, Corynebacterium striatum, Cronobacter sakazakii, Cutibacterium acnes, Enterobacter asburiae, Enterobacter cloacae, Enterobacter hormaechei, Enterobacter kobei, Enterobacter roggenkampii, Enterococcus durans, Enterococcus faecalis, Enterococcus faecium, Enterococcus hirae, Erwinia amylovora, Escherichia albertii, Escherichia coli, Eubacterium rectale, Faecalibacterium prausnitzii, Flavobacterium psychrophilum, Francisella tularensis, Gardnerella vaginalis, Haemophilus influenzae, Helicobacter pylori, Klebsiella aerogenes, Klebsiella grimontii, Klebsiella michiganensis, Klebsiella oxytoca, Klebsiella pneumoniae, Klebsiella quasipneumoniae, Klebsiella variicola, Lacticaseibacillus paracasei, Lacticaseibacillus rhamnosus, Lactiplantibacillus plantarum, Lactobacillus crispatus, Lactobacillus helveticus, Lactococcus lactis, Legionella pneumophila, Leptospira borgpetersenii, Leptospira interrogans, Ligilactobacillus salivarius, Limosilactobacillus reuteri, Listeria innocua, Listeria monocytogenes, Listeria seeligeri, Mannheimia haemolytica, Microcystis aeruginosa, Moraxella catarrhalis, Mycobacterium avium, Mycobacterium tuberculosis, Mycobacteroides abscessus, Mycoplasma bovis, Mycoplasma pneumoniae, Neisseria gonorrhoeae, Neisseria meningitidis, Oenococcus oeni, Parabacteroides distasonis, Parcubacteria group, Pasteurella multocida, Patescibacteria group, Phocaeicola vulgatus, Prevotella copri, Priestia megaterium, Proteus mirabilis, Pseudomonas aeruginosa, Pseudomonas fluorescens, Pseudomonas putida, Pseudomonas savastanoi, Pseudomonas stutzeri, Pseudomonas syringae, Pseudomonas viridiflava, Ralstonia solanacearum, Rhizobium leguminosarum, Salmonella enterica, SAR202 cluster,

	Serratia marcescens, Shigella boydii, Shigella dysenteriae, Shigella flexneri, Shigella sonnei, Sinorhizobium meliloti, Staphylococcus argenteus, Staphylococcus aureus, Staphylococcus capitis, Staphylococcus chromogenes, Staphylococcus epidermidis, Staphylococcus haemolyticus, Staphylococcus hominis, Staphylococcus pseudintermedius, Staphylococcus saprophyticus, Stenotrophomonas maltophilia, Streptococcus agalactiae, Streptococcus dysgalactiae, Streptococcus equi, Streptococcus mitis, Streptococcus mutans, Streptococcus oralis, Streptococcus pneumoniae, Streptococcus pseudopneumoniae, Streptococcus pyogenes, Streptococcus salivarius, Streptococcus suis, Thaumarchaeota archaeon, Thermoplasmata archaeon, Vibrio anguillarum, Vibrio cholerae, Vibrio parahaemolyticus, Vibrio vulnificus, Xanthomonas arboricola, Xanthomonas citri, Xanthomonas oryzae, Xanthomonas perforans, Xylella fastidiosa, Yersinia enterocolitica, Yersinia pestis
