# Supplementary figures and images for: Species-Specific Quality Control, Assembly and Contamination Detection in Microbial Isolate Sequences with AQUAMIS
Source: Genes (Basel). 2021 Apr 26;12(5):644. doi: 10.3390/genes12050644 (PMC8145556; doi:10.3390/genes12050644)

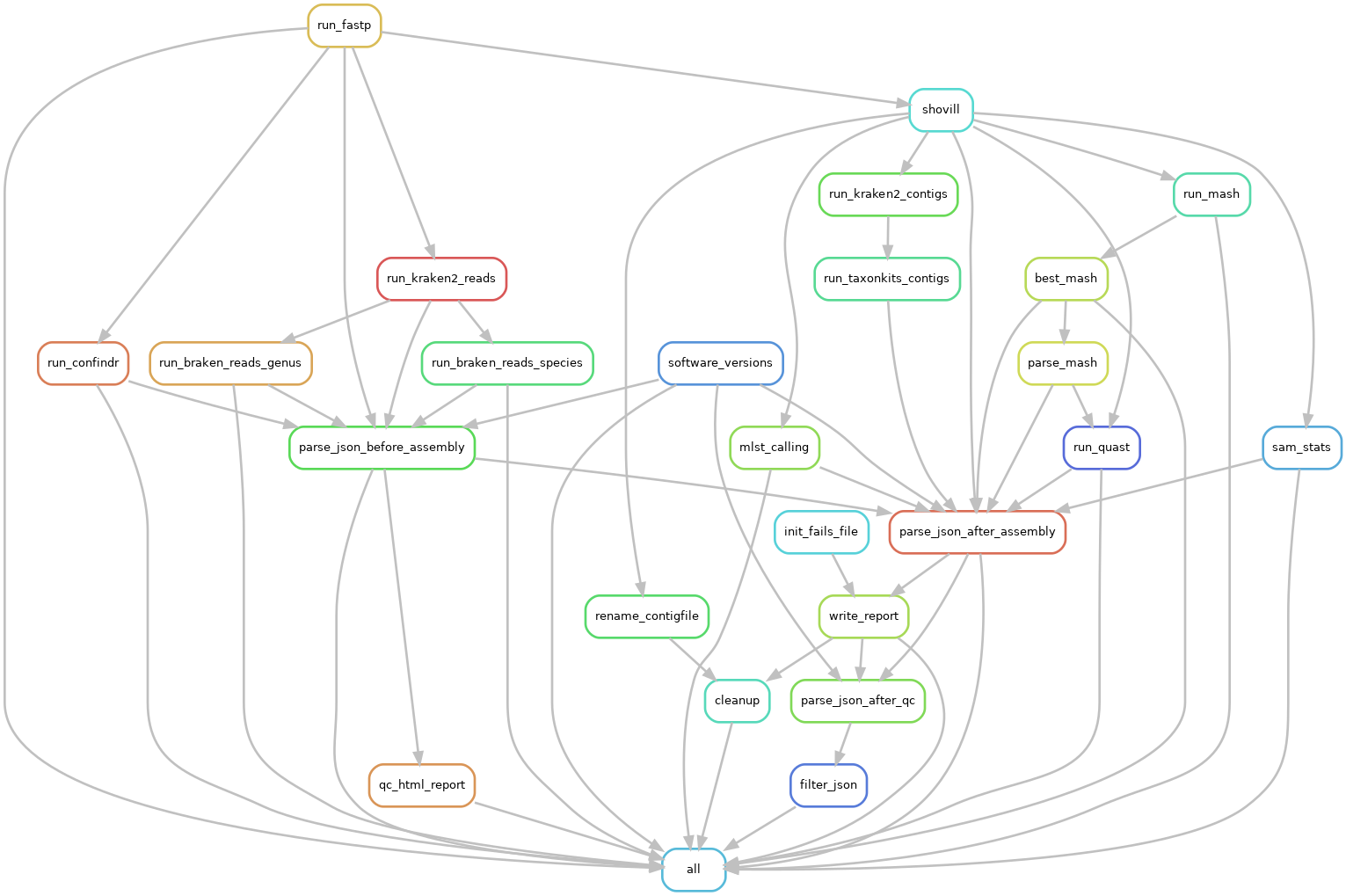

Supplement: Supplementary file 1 [file genes-12-00644-s001.zip › Supplementary Figure S1_dag.png]

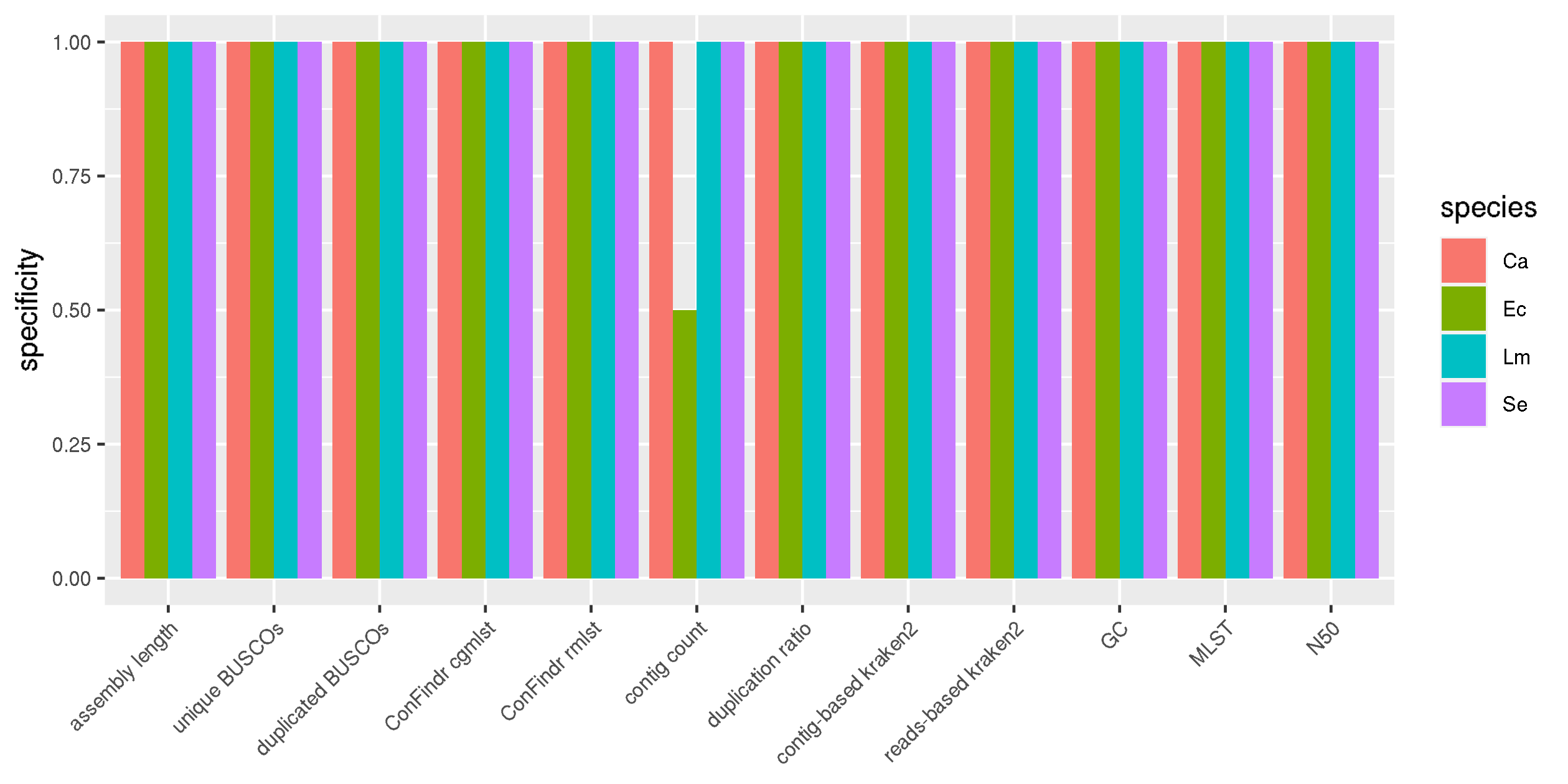

Supplement: Supplementary file 1 [file genes-12-00644-s001.zip › Supplementary Figure S2_prediction_self_byspecies.png]

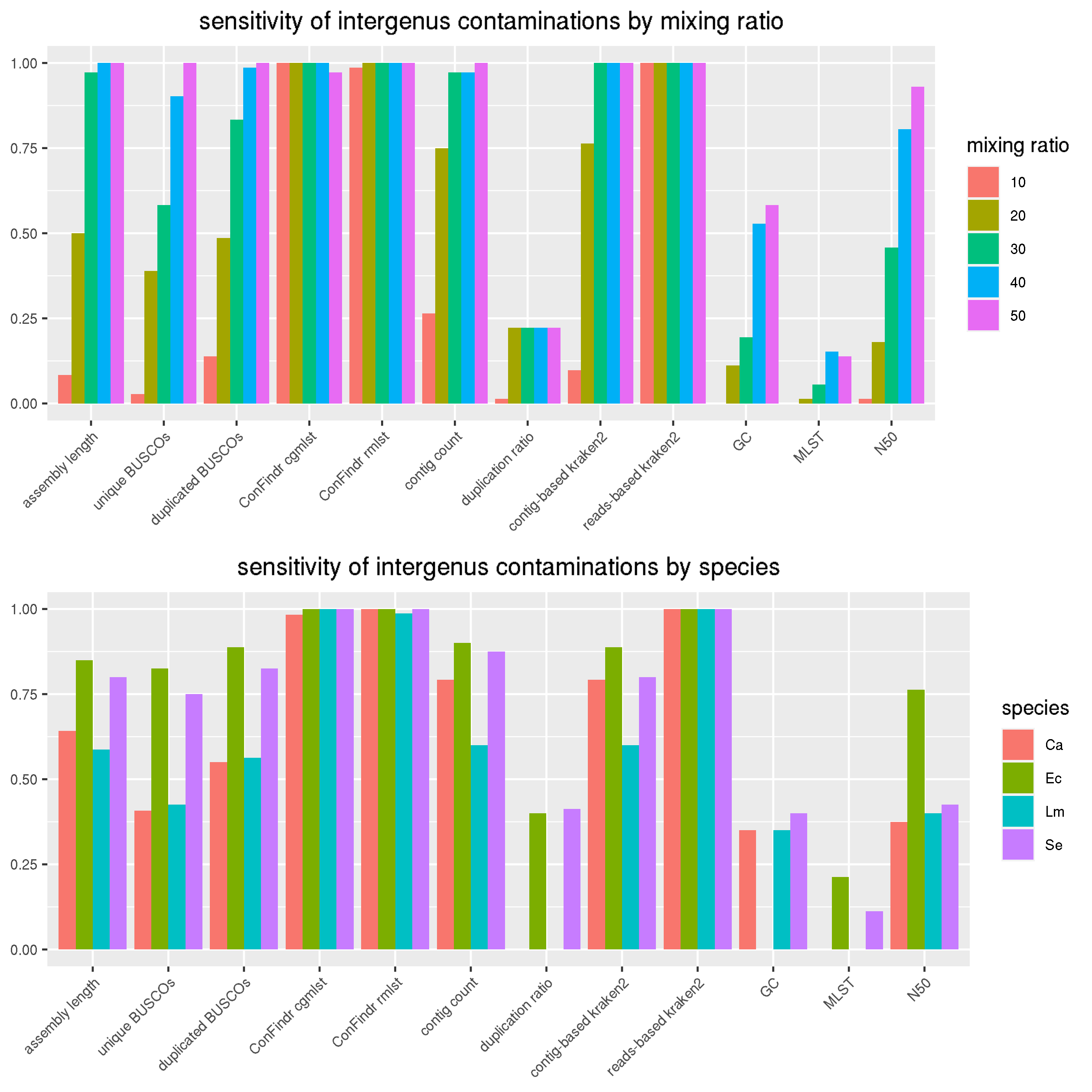

Supplement: Supplementary file 1 [file genes-12-00644-s001.zip › Supplementary Figure S3_prediction_inter_combi.png]

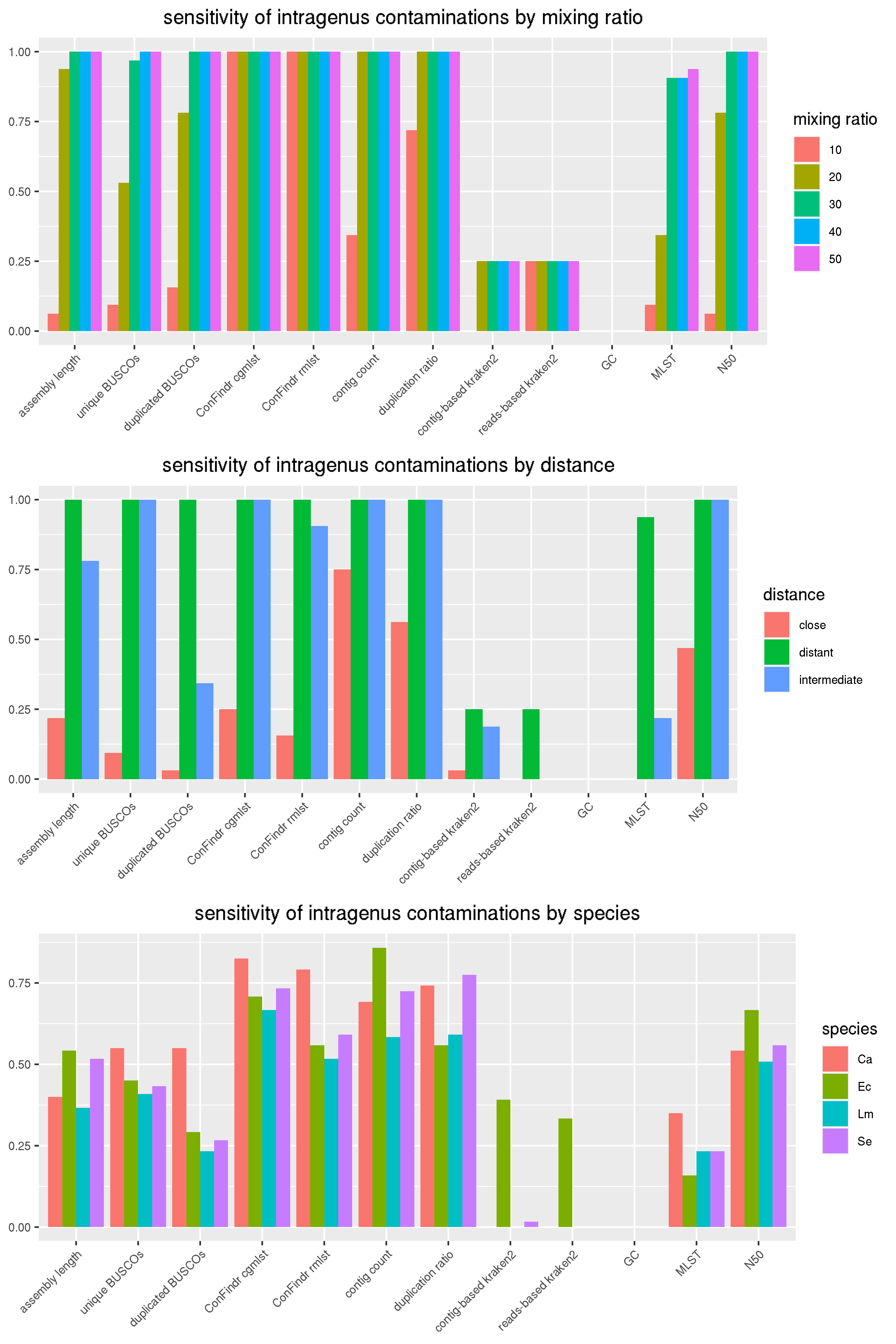

Supplement: Supplementary file 1 [file genes-12-00644-s001.zip › Supplementary Figure S4_prediction_intra_combi.png]

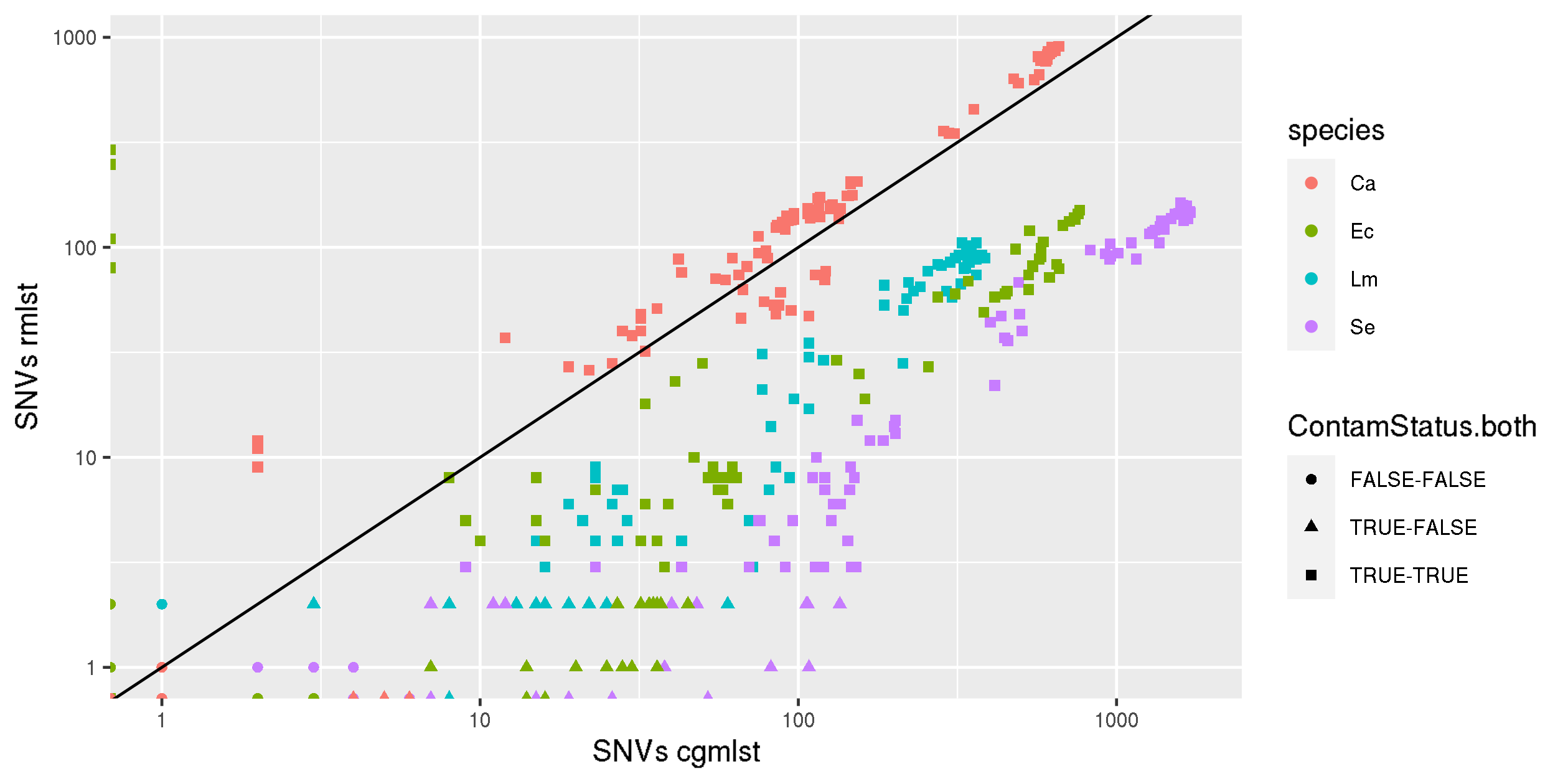

Supplement: Supplementary file 1 [file genes-12-00644-s001.zip › Supplementary Figure S5_confindr_cgmlst_vs_rmlst.png]
